# Drug Reaction With Eosinophilia and Systemic Symptoms (DRESS) Syndrome Induced by Primary Anti-tubercular Medication: A Case Report

**DOI:** 10.7759/cureus.50753

**Published:** 2023-12-18

**Authors:** Raymond Haward, Rachel Haward, JV Pranav Sharma

**Affiliations:** 1 Internal Medicine, Vydehi Institute of Medical Sciences and Research Centre, Bangalore, IND; 2 Internal Medicine, Kurunji Venkatramana Gowda (KVG) Medical College & Hospital, Sullia, IND; 3 General Surgery, Adesh Medical College and Hospital, Shahbad, IND

**Keywords:** severe eosinophilia, corticosteroids, systemic toxicity, drug allergy recording, anti-tuberculosis therapy, drug reaction with eosinophilia and systemic symptoms (dress) syndrome

## Abstract

Drug reaction with eosinophilia and systemic syndrome (DRESS) is a life-threatening hypersensitivity reaction of the skin and visceral organs caused by exposure to certain drugs, often with a latency period of two to eight weeks. A 20-year-old man, previously diagnosed with pulmonary tuberculosis (TB) one month ago and receiving treatment with isoniazid, rifampicin, pyrazinamide, and ethambutol (HRZE regimen), presented with symptoms including a maculopapular rash, fever, elevated transaminase levels, an increased white blood cell count with eosinophilia, hepatomegaly, and lymphadenopathy. The patient experienced recovery upon cessation of drug use and was administered corticosteroids and supportive therapeutic interventions. Individuals diagnosed with pulmonary TB who are undergoing treatment with first-line anti-tubercular medications have a heightened susceptibility to DRESS. The timely identification and cessation of the offending agent can effectively mitigate mortality.

## Introduction

Drug reaction with eosinophilia and systemic symptoms (DRESS) is a life-threatening hypersensitivity reaction of the skin and visceral organs caused by exposure to certain drugs, often with a latency period of two to eight weeks [1]. Drugs such as anti-epileptics, antibiotics including anti-tuberculars, antiretroviral agents, non-steroidal anti-inflammatory drugs (NSAIDs), and sulphonamides are frequently encountered as causes of DRESS [2].

Additionally, it affects both the organs within the body as well as the skin that covers them. Skin lesions encompass a range of manifestations, including an erythematous rash that extends over a substantial area of the body, a maculopapular rash, skin edema, exfoliation, and the presence of vesicles. In cases of significant severity, the Nikolsky sign may manifest as the detachment of blisters [3]. It is imperative to acknowledge that the cutaneous manifestations observed in DRESS syndrome represent only one facet of this clinical entity.

Individuals diagnosed with DRESS may also manifest symptoms affecting other organ systems such as the liver, kidney, lung, heart, and brain [2, 3]. The involvement of visceral organs in this condition is associated with heightened rates of morbidity and mortality. The global mortality rate is approximately 10% [3, 4].

The prevalence of DRESS remains uncertain, with an approximate population risk ranging from 1 in 1000 to 10,000 instances of drug exposure [2]. Genetics and immunological mechanisms are hypothesized to underlie the causes of DRESS. This syndrome occurs due to a type 4 hypersensitivity reaction triggered by the offending drugs [5].

DRESS is more prevalent among patients with autoimmune diseases. Helper T cells, recruited in response, release IL-5, leading to an increase in eosinophils, while cytotoxic CD8 T cells cause manifestations in the organs and integumentary system [6]. A correlation has been observed with viral infections such as human herpesvirus 6 (HHV-6), Epstein-Barr virus, and herpes reactivation [5]. Identifying the culprit drug is crucial for effective treatment [1].

The vignette describes a 20-year-old male who has been diagnosed with pulmonary tuberculosis (TB) for one month. The patient's treatment plan includes the isoniazid, rifampicin, pyrazinamide, and ethambutol (HRZE) regimen.

## Case presentation

**Figure 1:**
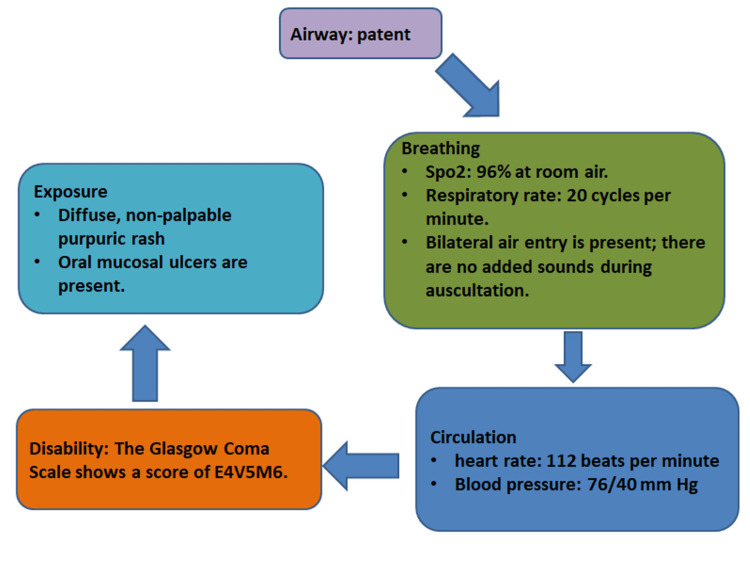
Initial Assessment in the Emergency Room, Comprising Airway, Breathing, Circulation, Neurological Status (Disability), and Exposure

A 20-year-old male patient previously diagnosed with pulmonary TB one month ago and initiated on an HRZE regimen was presented to the emergency department with complaints of nausea, generalized weakness, fever (38.2°C), and a rash persisting for two weeks. The initial assessment, or primary survey, is a crucial step in evaluating a patient's condition (Figure 1).

**Figure 2:**
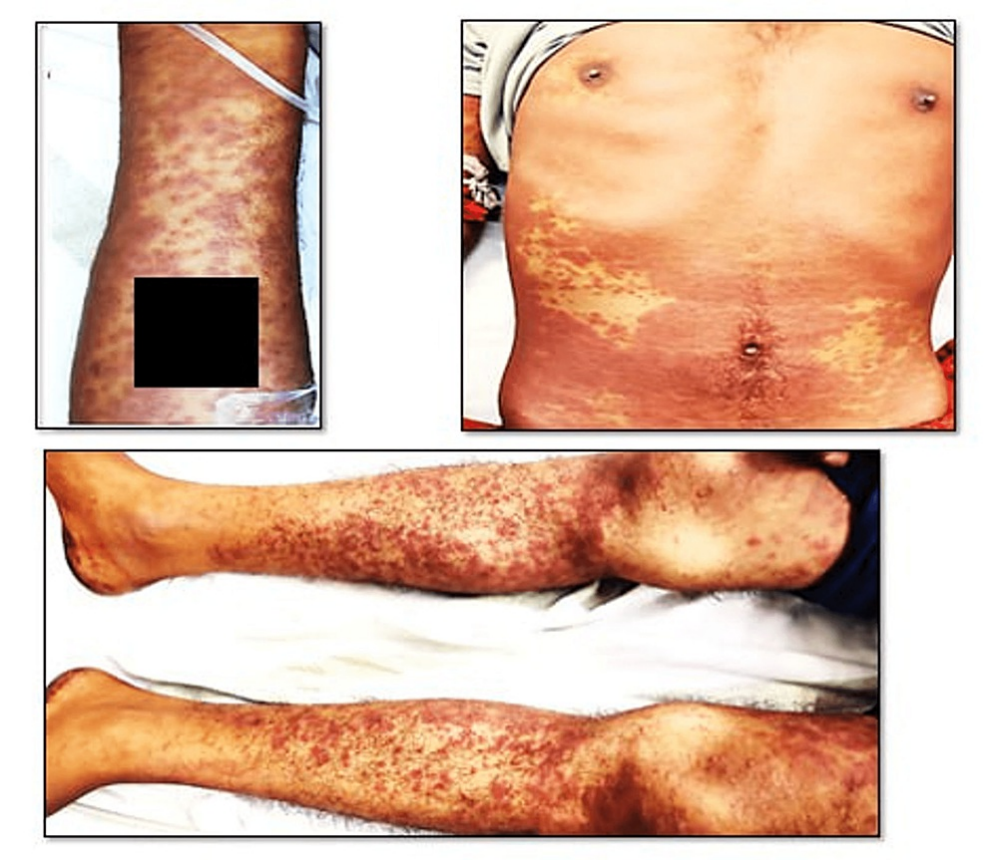
Scoring 1 in RegiSCAR Criteria for Skin Rash Extending Over 50% of Body Surface Area RegiSCAR: Registry of Severe Cutaneous Adverse Reactions

Upon conducting an inquiry with the patient, he provided an account of experiencing two weeks of fever. He posited that this occurrence could potentially be attributed to his TB condition and the HRZE regimen he had been prescribed. The onset of the rash occurred on the left hand around two weeks ago and thereafter spread to encompass the entirety of the body (Figure 2).

There was a lack of documented medical history pertaining to the utilization of alternative pharmaceutical substances. In April 2023, the patient received a diagnosis of pulmonary TB and was subsequently initiated on an HRZE regimen. The dosage regimen included isoniazid at a dose of 600 mg, rifampicin at a dose of 450 mg, pyrazinamide at a dose of 1500 mg, and ethambutol at a dose of 1200 mg.

**Figure 3:**
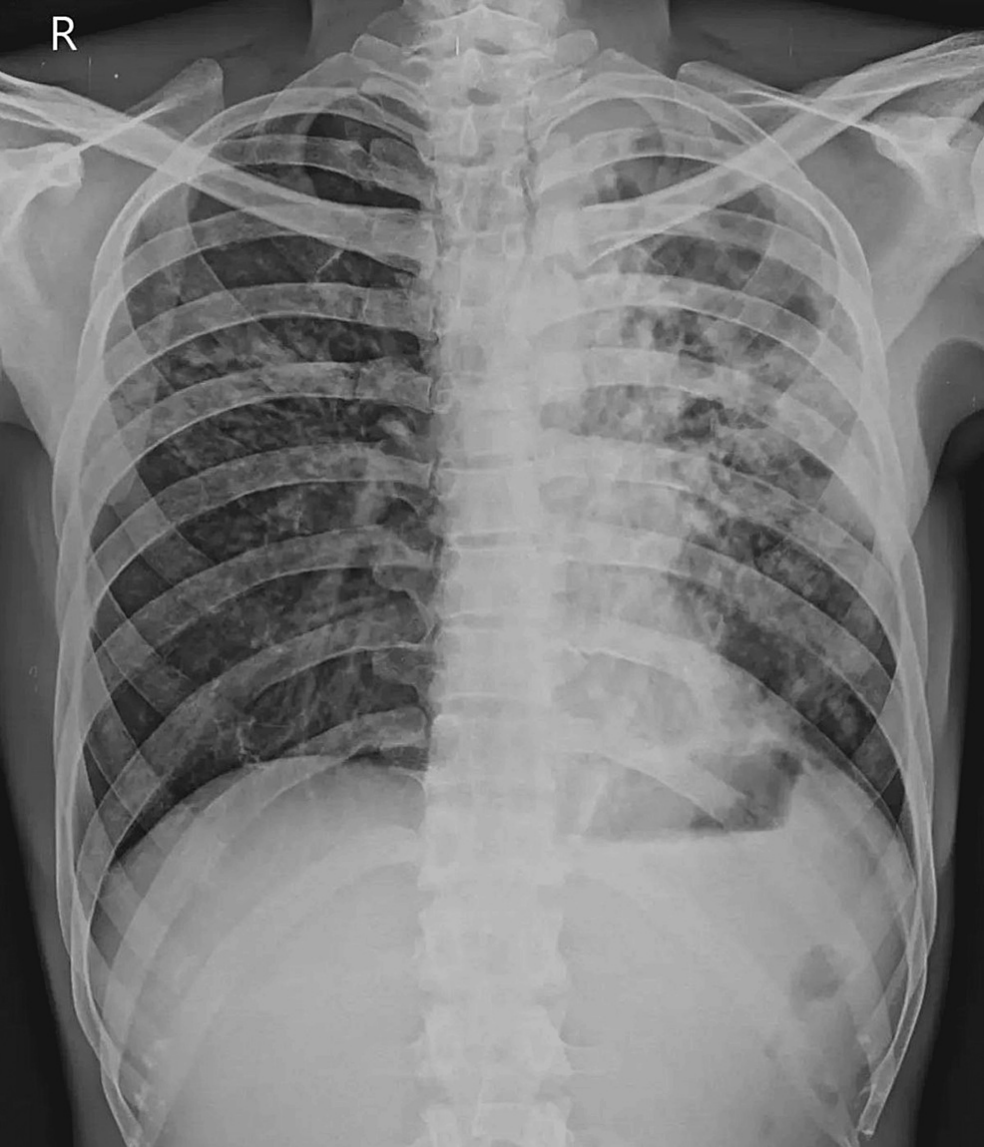
Pulmonary Tuberculosis Patient's Posterior-Anterior View Chest X-ray The radiological examination revealed coarse nodular infiltrates with air bronchogram and a dense area of consolidation in the right upper and mid zones, without evident volume loss. Additionally, coarse nodular infiltrates with air bronchogram, areas of breakdown, and dense consolidation were observed, accompanied by volume loss and a substantial cavitated region in the left upper, mid, and lower zones. These radiographic characteristics strongly suggest a diagnosis of tuberculosis.

Upon examination, the patient presented with symptoms of pallor, icterus, lymphadenopathy, and hepatomegaly. The results of the point-of-care tests indicated a blood glucose level of 75 mg/dL, an arterial blood gas analysis revealing metabolic acidosis, and a lactate level of 6.8 millimoles per liter. The systemic examination revealed an inferior vena cava measuring 1 cm and exhibiting collapsibility. The cardiac assessment indicated hyperdynamic activity, while the lung examination showed a bilateral A profile. The resuscitation procedure involved administering 8 mg of noradrenaline, diluted in 50 mL of normal saline, delivered at a rate of 4 mL per hour. The potential differential diagnoses for the observed symptoms at the specific period in question include septic shock with disseminated intravascular coagulation (DIC), adrenal shock, DRESS, meningococcemia, and thrombotic thrombocytopenic purpura (TTP). The chest X-ray revealed the presence of pulmonary TB (Figure 3).

**Figure 4:**
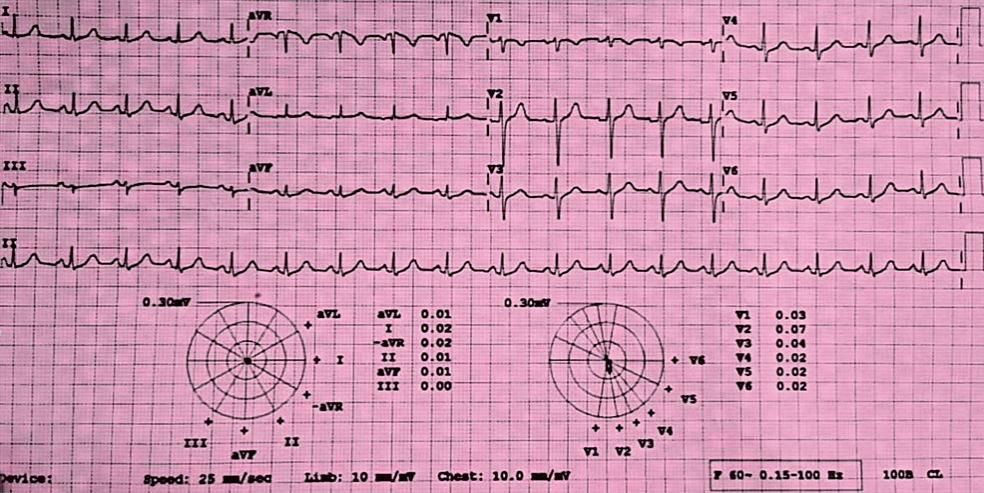
Electrocardiogram on Arrival at the Emergency Department Suggests Sinus Tachycardia

The ECG revealed sinus tachycardia (Figure 4).

Our blood tests showed that there was eosinophilia, transaminitis, and high levels of C-reactive protein (CRP) (Table 1).

**Table 1:** Laboratory Investigation Results PT: prothrombin time; INR: international normalized ratio; PTT: partial thromboplastin time; SGOT: serum glutamic oxaloacetic transaminase (also known as AST, aspartate aminotransferase); SGPT: serum glutamic pyruvic transaminase (also known as ALT, alanine aminotransferase); HIV: human immunodeficiency virus; HCV: hepatitis C virus; HBsAg: hepatitis B surface antigen.

Laboratory Investigation	Patient Value	Reference Unit	Reference Value
Hemoglobin	11	g%	12–16
White blood cell (WBC) count	17,050	cells/mm^3^	4000–11,000
Neutrophil%	40	%	50–70
Lymphocyte%	36.7	%	20–40
Eosinophil%	22.3	%	0.5–5
Basophil%	1	%	0–1
Absolute eosinophil count	3800	mm^3^	<350
Blood urea	37	mg/dL	10–50
Blood urea nitrogen (BUN)	17.3	mg/dL	4.7–23.3
Serum creatinine	0.7	mg/dL	0.6–1.2
Sodium	138	mmol/L	136–145
Potassium	4.9	mmol/L	3.5–5.1
Chloride	104	mmol/L	98–107
Anion gap	15	mmol/L	8–16
Glucose	75	mg/dL	70–110
Calcium	9	mg/dL	8.4–10.5
PT	21.5	seconds	9.5–12
INR	1.65		<1.1
PTT	32	s	21–32.0
Lactate	6.8	mmol/L	0.5–2.2
D dimer	2.1	mg/L	< 0.50
Total bilirubin	1.6	mg/dL	0.2–1.2
Direct bilirubin	0.7	mg/dL	0.1–0.4
Total protein	7	g/dL	6.2–8
Globulin	3.4	g/dL	1.8–3.5
Albumin	3.6	g/dL	3.5–5.5
SGOT	781	U/L	6–46
SGPT	642	U/L	8–49
Alkaline phosphatase	171	U/L	35–104
C-reactive protein (CRP)	96.5	mg/L	<0.4
HBsAg	Negative		
HIV-1 antibody	Negative		
HCV antibody	Negative		

Upon conducting the Registry of Severe Cutaneous Adverse Reactions (RegiSCAR) scoring, the resulting score of 6 indicated the presence of DRESS syndrome in the patient (Table 2).

**Table 2:** RegiSCAR Criteria [7] A final score below 2 indicates no case, while a score between 2 and 3 suggests a possible case. A score ranging from 4 to 5 indicates a probable case, and a score above 5 confirms a definite case. Evaluation of other potential causes includes antinuclear antibody, blood culture, serology for hepatitis A virus, hepatitis B virus, hepatitis C virus, and chlamydia/mycoplasma. If none of these tests yield positive results and at least three of the aforementioned criteria are negative, a score of 1 is awarded. RegiSCAR: European Registry of Severe Cutaneous Adverse Reaction; DRESS: drug reaction with eosinophilia and systemic symptoms; BSA: body surface area.

Clinical Parameters	Score			
	-1	0	1	2
Fever (≥38.5°C)	No/Unknown	Yes		
Enlarged lymph nodes		No/Unknown	Yes	
Eosinophilia		No (0–699 cells or <10%)/Unknown	Yes (700–1499 cells, or 10–19.9%)	Yes (≥1500 cells or ≥20%)
Atypical lymphocytes		No/Unknown	Yes	
Skin rash extent: ≥50% of BSA		No/Unknown	Yes	
Rash suggestive of DRESS	No	Unknown	Yes	
Skin biopsy suggestive of DRESS	No	Yes/Unknown		
Organ involvement		No	Yes (1 organ)	Yes (≥2 organs)
Disease duration: ≥15 days	No/Unknown	yes		
Exclusion of other causes		No/Unknown	Yes	

This conclusion stemmed from factors including enlarged lymph nodes (1 point), eosinophilia ≥20% (2 points), skin rash suggesting DRESS covering over 50% of the body surface area (2 points), and liver involvement (1 point), totaling six points. The antitubercular regimen suspected of causing harm was discontinued. The patient was then initiated on the intravenous administration of 60 mg of methylprednisone in conjunction with supportive interventions.

Upon further evaluation, it was observed that the patient's shock condition showed improvement, and the metabolic acidosis was successfully treated by the process of lactate clearance. Following the transfer of the patient to the ward, a regimen of intravenous methylprednisone at a dosage of 1.5 mg/kg/day was administered for seven days. Additionally, oral methylprednisone at a dosage of 4 mg/day was prescribed. The patient responded positively to the steroid treatment. Patch tests confirmed Rifampicin and Isoniazid as the culprit medications. The patient received a topical application of liquid paraffin lotion and betamethasone cream to alleviate symptoms until there was improvement of skin lesions, normalization of liver function tests, and a reduction of eosinophils to optimal levels. The individual was discharged from medical care on the fifteenth day following the resolution of the skin eruption, which resulted in the development of hyperpigmentation. The patient was referred to the Department of Pulmonary Medicine for the management of TB and subsequently initiated a regimen of second-line anti-tubercular medications. The subsequent visit, occurring one week later, revealed the absence of any resurgence of the reaction. The therapy involving ethambutol, linezolid, moxifloxacin, and amikacin was deemed successful since no adverse reactions were noted.

## Discussion

The definition of DRESS, first used by Bocquet [8], is a severe cutaneous reaction with fever and systemic manifestations that endangers the patient's life. The clinical presentation includes the presence of a maculopapular rash, a fever exceeding 38°C, increased levels of transaminase, a raised white blood cell count with an increase in eosinophils and atypical lymphocytes, and the presence of lymphadenopathy. The pathogenesis of DRESS syndrome encompasses various elements, including disrupted drug metabolism, immunological responses, and the correlation with reactivated herpes viruses [9]. Genetic variations in enzymes such as cytochrome P (CYP) 450 enzyme, N-acetyltransferase, and epoxide hydrolase, which play a crucial role in detoxifying intermediate drug metabolites, can result in the accumulation of these metabolites [9, 10]. This accumulation can trigger immune responses by activating CD4 and CD8 T cells [9, 10, 11]. Despite numerous culprit drugs, the precise genetic predisposition for DRESS syndrome remains unidentified. However, certain genetic links have emerged, particularly associations between specific HLA types and drug exposure [10]. Notable examples include the connection of Abacavir with DRESS in patients possessing HLA-B*5701, Allopurinol with HLA-B*5801 in Chinese ethnicity, along with Stevens-Johnson syndrome (SJS) and toxic epidermal necrolysis (TEN), and carbamazepine with HLA-DR3, HLA-DQ2, and HLA-A*3101 [9, 10].

An observed correlation exists between the presence of HHV6 virus in patients suffering from DRESS syndrome, indicated by tests revealing IgG against HHV6 and the identification of genetic material, alongside other viruses like Epstein-Barr virus, cytomegalovirus, and HHV7 [10]. Hypotheses suggest that viral reactivation either directly causes DRESS syndrome or that culprit drugs and subsequent viral reactivation lead to a cytokine storm [9]. However, the precise mechanism linking DRESS and virus activation and its significance still remains unclear [10]. Rifampicin has the highest frequency of causing DRESS among other TB medications, although cases also exist for ethambutol, isoniazid, pyrazinamide, streptomycin, fluoroquinolones, and para-aminosalicylic acid [12]. The clinical manifestations should encompass both cutaneous and visceral organs in a typical DRESS syndrome.

The clinical vignette presented symptoms including fever, a widespread rash characterized by macules and papules, raised levels of liver enzymes, an increased white blood cell count with elevated eosinophils, and the presence of enlarged lymph nodes together with an enlarged liver. The RegiSCAR criteria were created to distinguish DRESS from other skin-related adverse reactions, such as SJS or toxic epidermal necrolysis [11]. The patient exhibited a RegiSCAR score of 6, indicative of the suggested diagnosis. The treatment protocol entails discontinuing the medicine responsible for DRESS and administering intravenous corticosteroids and fluids in the emergency department.

TB is considered endemic in India, with a significant proportion of identified TB cases worldwide originating from this country, accounting for around 28% of the global TB burden in 2021 [13]. ATT medications, such as isoniazid, rifampicin, streptomycin, and pyrazinamide, are known for their potential to induce DRESS syndrome. However, they also function as both preventive and therapeutic agents against TB transmission and infection. After consultation with the respiratory department, the patient's treatment was modified to include second-line ATT. On subsequent follow-up, there was a complete resolution of the symptoms.

## Conclusions

A 20-year-old Indian male on HRZE reported nausea, weakness, fever, and rash for two weeks. He showed maculopapular rash, a fever of 38.2°C, increased transaminase, leucocytosis with eosinophilia, hepatomegaly, and lymphadenopathy. The RegiSCAR criteria yielded a score of 6, confirming the diagnosis of DRESS. Supportive treatment and glucocorticoids managed the symptoms. The patient was subsequently prescribed ethambutol, linezolid, moxifloxacin, and amikacin for pulmonary TB, as rifampicin and isoniazid were identified as the culprit drugs. Follow-up demonstrated complete relief of symptoms.
